# Temporal transcriptomic and lipidomic analysis reveals multi-omics dynamic profiles of *B. napus* seed germination

**DOI:** 10.1186/s12864-026-13202-y

**Published:** 2026-07-20

**Authors:** Bo Zhang, Junyan Wu, Li Ma, Haiqing Liu, Wancang Sun, Jianfeng Duan

**Affiliations:** 1 Gansu Agricultural University Qingyang Winter Rapeseed Science and Technology Yard, Qingyang, 745000 China; 2 Qingyang Urban and Rural Construction Development Investment Group Co., Ltd, Qingyang, 745000 China; 3 State Key Laboratory of Aridland Crop Science, College of Agronomy, Gansu Agricultural University, Lanzhou, 730070 China; 4 Longdong University, Qingyang, 745000 China; 5 Qingyang Agricultural Ecological Environmental Protection Management Station, Qingyang, 745000 China

**Keywords:** Lipids, Seed germination, Transcriptomic and Lipidomic, *B. napus.*L

## Abstract

**Background:**

Lipids represent the major storage reserve in Brassica napus seeds. During germination, lipid mobilization delivers indispensable energy to support seedling establishment, thereby profoundly influencing germination vigor and subsequent seedling growth capacity. Although lipid mobilization is fundamentally required for successful rapeseed germination, the temporal coordination between transcriptional reprogramming and lipid metabolic conversion remains largely elusive, and the underlying molecular regulatory network remains to be systematically deciphered.

**Results:**

We conducted an integrated transcriptomic and lipidomic analysis on dry *B. napus* seeds and germinating seeds at 6, 12, 24, and 48 h after imbibition. The results revealed distinct stage-specific characteristics of gene expression and lipid metabolism during germination. In the early imbibition stage, differentially expressed genes (DEGs) were primarily enriched in biological processes related to water transport, stress response, and signal transduction, whereas significant changes in lipid metabolism were observed to be relatively delayed. During the initiation of germination, triacylglycerols (TGs) underwent rapid degradation, accompanied by a significant up-regulation of genes involved in the β-oxidation and gluconeogenesis pathways. In the late germination stage, genes responsible for membrane lipid synthesis were sharply up-regulated, which induced extensive membrane lipid remodeling.

**Conclusion:**

This work represents the first systematic integration of transcriptomic and lipidomic data focusing on dynamic changes during the germination stage in *B. napus*, systematically illustrating the global molecular and lipid metabolic features of rapeseed seeds across sequential imbibition stages. It further delineates stage-specific expression patterns of key functional genes and lipid metabolites throughout germination. Collectively, these results advance our comprehensive understanding of the regulatory networks controlling rapeseed seed germination, and offer reliable theoretical references and candidate gene resources for breeding high-yield and high-quality *B. napus* varieties.

**Supplementary Information:**

The online version contains supplementary material available at https://doi.org/10.1186/s12864-026-13202-y.

## Background

Seed germination, a key early event in the plant life cycle, is distinct from the subsequent phase of seedling establishment, which involves the transition from heterotrophic to autotrophic growth and ultimately determines field crop emergence and stand uniformity [1–3]. Seed germination is a sophisticated biological process synergistically regulated by the interplay of hormone signaling, metabolism and genetic networks [4]. *B. napus* L. is one of the most important oilseed crops worldwide, with its seeds containing approximately 40% oil on a dry weight basis, serving as a major source of edible vegetable oil, animal feed, and renewable bioenergy feedstocks [5–7]. In contrast to cereal crops, which rely predominantly on starch reserves to fuel germination, oilseed species such as *B. napus* depend primarily on the mobilization of stored triacylglycerols (TGs) within lipid droplets as the principal carbon and energy source for germination and early seedling growth [8, 9]. Dynamic lipid degradation, transport and transformation processes are key prerequisites for successful seed germination and the seamless establishment of seedlings [10].

Triacylglycerols (TGs) represent the primary storage lipid in *B. napus* seeds and play two core roles during germination. On one hand, stored lipids are catabolized via a cascade of metabolic reactions to generate energy substrates, which supply the energy required for seed imbibition, radicle emergence, and early seedling growth [11]. Conversely, intermediate lipid metabolites serve as precursors for cell membrane synthesis and remodeling. These pathways underpin the preservation of cellular structural integrity and membrane fluidity, thereby supporting core physiological events including cell proliferation and differentiation [12].

The molecular regulatory mechanisms controlling seed germination have not been fully elucidated. A set of core pathways and functional genes have been documented to participate in this process. In phytohormone signaling pathways, GA activates the transcription of germination-related genes and relieves seed dormancy, while ABA suppresses germination [13–15]. Within lipid metabolic cascades, lipid hydrolysis, β-oxidation and gluconeogenesis function in concert to mobilize storage lipids into energy substrates and carbon skeletons, thereby supporting seed germination [16, 17].

In this study, we collected samples at successive time points and integrated transcriptomic and lipidomic profiling to dissect the temporally coordinated dynamic patterns of gene expression and lipid metabolic signatures during seed imbibition and germination of *B. napus*. Our results may offer preliminary theoretical references for the genetic improvement of germination-associated traits in *B. napus*. However, the regulatory mechanisms through which stage-specific core regulatory genes and lipid metabolites govern seed germination progression require further systematic exploration.

## Methods

### Plant materials and sample collection

In this study, *B. napus* seeds (cultivar LY80) with a genetically stable background. All water content determination, morphological observation, RNA-seq and lipidomic profiling used seeds harvested from the same single batch of LY80 plants, dried, sealed and stored under unified 4 ℃ conditions, avoiding batch variation. Prior to germination, plump, intact seeds of uniform size were disinfected with 75% ethanol, rinsed thoroughly with sterile water for five times, and then placed in Petri dishes lined with double layers of filter paper for germination in a constant-temperature incubator at 25 ℃ in the dark.

The time points of 0, 6, 12, 24, and 48 h were selected based on preliminary morphological observations and water uptake kinetics of LY80 rapeseed seeds. At 0 h, dry seeds were set as the initial dormant control with no water absorption. The 6 h time point corresponded to the peak of rapid water imbibition, during which seeds swelled without seed coat cracking or radicle emergence, representing the early imbibition stage. At 12 h, water absorption entered a slow plateau phase; embryos expanded and seed coats softened while remaining intact, marking the mid imbibition pre-germination stage. The 24 h time point served as the critical germination transition stage, when radicles pierced through the seed coat. At 48 h, radicles elongated rapidly alongside hypocotyl extension, accompanied by full rupture of the seed coat.

Samples were collected at 0 h, 6 h, 12 h, 24 h and 48 h after seed imbibition, each independent biological replicate contained exactly 90 uniform-sized intact plump seeds, and the identical seed number was applied to all time points. Each independent biological replicate consisted of approximately 0.2 g of mixed seeds, and three biological replicates were prepared for each time point. Before grinding and freezing, seeds with asynchronous germination, cracked seed coat or abnormal embryo development were manually eliminated. All samples were snap-frozen in liquid nitrogen and stored at -80 ℃ for subsequent transcriptomic and metabolomic analyses.

### RNA-sequencing (RNA-seq) analysis

In this study, Total RNA was isolated and checked for integrity using an Agilent 2100 Bioanalyzer. cDNA libraries were constructed and sequenced on an Illumina NovaSeq 6000 (high-throughput next-generation sequencing (NGS) platform to generate 150-bp paired-end reads. Clean reads were mapped to the *B. napus* reference genome (ZS11.v10) using HISAT2 software (HISAT2 v2.2.1), and DEGs were identified using DESeq2 (DESeq2 v1.42.0) with Benjamini–Hochberg correction. Genes with padj < = 0.05 and |log2 (fold change)| >= 1 were defined as significantly DEGs in this study. Before differential expression analysis, low-expression genes with average FPKM < 0.5 across all biological samples were filtered out; a total of 34,689 high-confidence expressed genes were retained for downstream analysis. KEGG (Kyoto Encyclopedia of Genes and Genomes) annotations, the enrichment analysis of differentially expressed genes (DEGs) were performed using cluster Profiler (3.8.1).

Weighted gene co-expression network analysis (WGCNA, v1.6.6, R) was used to identify lipid metabolism-related co-expression modules in germinating *B. napus* seeds. After filtering low-expression genes, 34,690 genes were retained for network construction. The pickSoftThreshold function identified β = 10 as the optimal soft threshold, which achieved a scale-free topology fit R² > 0.8 with acceptable mean connectivity. Gene correlations were converted into an adjacency matrix and then a topological overlap matrix (TOM). Genes were hierarchically clustered via dynamic tree cutting (minimum module size = 30) based on 1-TOM distances. Redundant modules with eigengene dissimilarity < 0.25 were merged.

### Lipid extraction and UHPLC-MS/MS analyses

Total lipids were extracted using a modified methyl tert-butyl ether (MTBE) method. Briefly, 100 mg of seed powder was homogenized in 0.75 mL of methanol, mixed with 2.5 mL of MTBE, and agitated for 60 min at room temperature. Phase separation was induced by adding 0.625 mL of ultrapure water, and the mixture was subsequently left undisturbed for 10 min to allow the layers to separate completely [18]. Equal aliquots from all test samples were mixed to prepare pooled QC samples, which were injected every 10 samples during LC-MS acquisition to monitor system stability. UHPLC-MS/MS analyses were conducted using a Vanquish UHPLC system (Thermo Fisher Scientific, Germany) coupled to an Orbitrap Q Exactive™ HF mass spectrometer (Thermo Fisher Scientific, Germany). Chromatographic separation was performed on a Thermo Accucore C30 column (150 × 2.1 mm, 2.6 μm) with a 20-min linear gradient at a flow rate of 0.35 mL/min; the column temperature was maintained at 40 °C. Mobile phase A consisted of acetonitrile–water (60:40, v/v) with 10 mM ammonium acetate and 0.1% formic acid; mobile phase B was acetonitrile–isopropanol (10:90, v/v) with the same additives. The gradient elution was as follows: 30% B (0–2 min), 43% B (2–5 min), 55% B (5.1 min), 70% B (5.1–11 min), 99% B (11–16 min), and return to 30% B for re-equilibration at 18.1 min .

The mass spectrometer was operated in both positive and negative ionization modes. Key MS parameters were set as follows: sheath gas, 40 psi; sweep gas, 0 L/min; auxiliary gas flow rate, 10 L/min (positive) / 7 L/min (negative); spray voltage, 3.5 kV; capillary temperature, 320 °C; heater temperature, 350 °C; S-Lens RF level, 50; mass scan range, 114–1700 m/z; full scan AGC target, 3 × 10⁶; normalized collision energies, 22, 24, and 28 eV; maximum injection time, 100 ms; isolation window, 1 m/z; MS² AGC target, 2 × 10⁵; and dynamic exclusion duration, 6 s.

### Lipidomic data analysis

Raw UHPLC-MS/MS data were processed using LipidSearch software for lipid annotation. To achieve reliable lipid annotation, we integrated three independent validation metrics instead of relying solely on database searching. We matched precursor exact masses within a mass tolerance below 5 ppm, detected subclass-specific diagnostic MS/MS fragment profiles, and aligned measured retention times against the internal LIPID MAPS library. Only lipid species fulfilling all three matching requirements were carried forward for subsequent quantitative analysis. Following annotation, lipid intensity tables were filtered to retain species detected in ≥ 70% of samples. Remaining missing values were imputed by k-nearest neighbor (kNN, k = 5) on log₂-transformed intensities; only lipid species passing all three annotation criteria were used in downstream quantitative analysis. Principal component analysis (PCA) and partial least squares discriminant analysis (PLS-DA) were conducted using metaX, a comprehensive software platform for metabolomic data processing. Univariate t-tests were applied to calculate *P*-values for each lipid metabolite. Lipids with VIP > 1, *P*-value < 0.05, and fold change ≥ 2 or ≤ 0.5 were defined as differential lipid metabolites. Volcano plots were generated based on log₂(fold change) and − log₁₀(*P*-value) to visualize differential lipids.

### RNA extraction and quantitative RT-PCR

Total RNA was extracted from 0.2 g rapeseed powder with RNAiso Plus. RNA purity was quantified using a NanoDrop 200, and 2 µg RNA was reverse-transcribed into cDNA. The *B. napus* β-Actin gene (GenBank accession: AF111812) was used as the internal reference gene for qRT-PCR detection. The full coding sequence of *BnActin* is provided above. The qPCR primer sequences of *BnActin* were as follows: forward primer 5′-CGAGGCTCCTCTTAACCCAAAGG-3′, reverse primer 5′-CACCAGAATCCAGCACAATACCG-3′. Gene-specific primers were designed using Primer 5 (Table S5). For each primer pair, standard curves were generated with serial dilutions of cDNA templates to calculate amplification efficiency, and all primer efficiencies ranged from 89% to 105%. Three biological and technical replicates were set, and relative expression levels were determined via the comparative 2 − ΔΔCt threshold method [19].

## Results

### Seed morphology and water content after imbibition

**Fig. 1:**
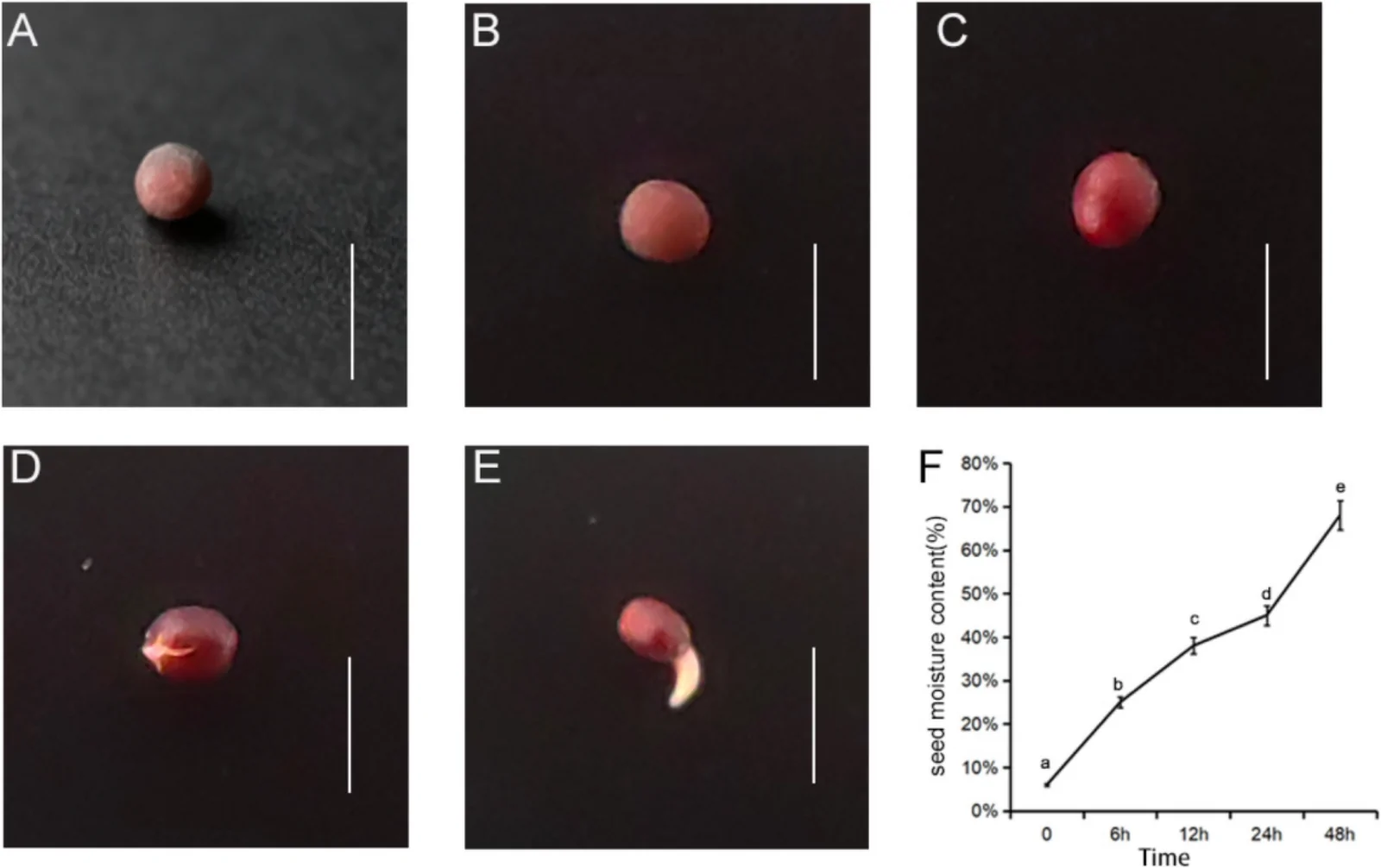
Morphological changes of *B. napus* seeds during seed imbibition. Panels (**A**–**E**) show seed phenotypes at 0 h, 6 h, 12 h, 24 h, and 48 h after imbibition, respectively. **F**, Comparison of water content in *B. napus* seeds at different imbibition stages. One-way ANOVA followed by Tukey’s multiple comparison test was performed for seed moisture content across time points. Different lowercase letters indicate significant differences at *P* < 0.05. Error bars represent the standard error of the mean

To clarify the temporal characteristics of *B. napus* seed imbibition and germination, we divided the process into sequential stages based on morphological observation and water content determination (Fig. 1). Dry seeds had a water content of 6.82%, which increased rapidly to 28.57% within 6 h, with slight seed swelling but intact seed coat and no radicle protrusion. From 6 to 12 h, water absorption slowed, reaching 39.24% at 12 h (Fig. 1C), accompanied by continued swelling, softened seed coat and embryo expansion without seed coat rupture. Between 12 and 24 h, water content stabilized at 35.11%–42.38% (Fig. 1D), with radicle protrusion and slight seed coat cracking. After 24 h, water content remained stable, with rapid radicle elongation to at 48 h, hypocotyl stretching and complete seed coat rupture (Fig. 1F). Temporal characteristics of seed imbibition lay a fundamental foundation for subsequent omics detection and analysis.

### Global dynamic changes of transcriptome during seed imbibition and germination

To systematically explore the transcriptional dynamics during *B. napus* seed imbibition and germination, transcriptome sequencing was performed on seed samples at 0 h, 6 h, 12 h, 24 h, and 48 h after imbibition, with three biological replicates per time point. Quality assessment revealed that the Q30 score for all sequencing libraries exceeded 97.36%, and the GC content ranged from 46.29% to 47.04%, indicating high sequencing accuracy and reliability, which met the requirements for subsequent bioinformatics analysis (Table 1). Meanwhile, transcriptome sequencing reads were aligned to the *B. napus* reference genome. The Total_map (≥ 91.75%) and Unique_map (≥ 84.59%) of all samples fell within a reasonable high-value range with good alignment efficiency, demonstrating high reliability of the sequencing data and its suitability for subsequent analyses. Principal Component Analysis (PCA) was adopted to assess the overall differences and repeatability of transcriptomic patterns among samples at different germination stages. The results showed that samples collected at the same time point clustered tightly, indicating reliable reproducibility across biological replicates. Samples from distinct germination stages were clearly separated along PC1, which explained 63.71% of the total transcriptional variation, while PC2 contributed an additional 15.69% of the total variation (Figure S1). Visually, dry dormant seeds (Im0) were distinctly separated from all imbibed samples along PC1, which suggests that the most prominent transcriptional shifts take place between dry seeds and early water-imbibed seeds. Such ordered sample separation along the principal axes implies progressive transcriptional remodeling throughout seed imbibition and germination, and the clear temporal grouping of samples provides a solid basis for downstream differential gene expression analysis.

**Table 1:** Quality and genome alignment statistics of transcriptome data

Sample	Clean_reads	Error_rate	Q20	Q30	GC_pct	Total_map	Unique_map
Im0R1	68,807,008	0.01	99.44	97.65	47.04	63,748,432(92.65%)	58,491,777(85.01%)
Im0R2	71,868,122	0.01	99.39	97.47	46.67	66,516,572(92.55%)	60,790,905(84.59%)
Im0R3	71,232,200	0.01	99.36	97.47	46.73	65,579,636(92.06%)	60,256,477(84.59%)
Im6R1	75,799,360	0.01	99.39	97.48	46.96	69,910,950(92.23%)	65,035,249(85.8%)
Im6R2	74,211,060	0.01	99.39	97.54	46.76	68,278,888(92.01%)	63,402,952(85.44%)
Im6R3	75,960,730	0.01	99.37	97.36	46.92	69,885,929(92.0%)	64,409,897(84.79%)
Im12R1	75,916,102	0.01	99.42	97.54	46.45	69,896,072(92.07%)	65,302,548(86.02%)
Im12R2	76,203,644	0.01	99.38	97.49	46.29	69,916,393(91.75%)	65,546,390(86.01%)
Im12R3	74,881,814	0.01	99.42	97.54	46.38	68,977,426(92.12%)	64,707,700(86.41%)
Im24R1	75,852,410	0.01	99.41	97.47	46.69	70,150,592(92.48%)	66,070,476(87.1%)
Im24R2	74,776,434	0.01	99.42	97.6	46.64	69,196,973(92.54%)	65,068,678(87.02%)
Im24R3	76,766,098	0.01	99.45	97.53	46.69	71,101,624(92.62%)	66,878,307(87.12%)
Im48R1	73,803,716	0.01	99.44	97.58	46.99	68,631,698(92.99%)	64,762,903(87.75%)
Im48R2	71,675,682	0.01	99.41	97.53	46.96	66,525,494(92.81%)	62,620,014(87.37%)
Im48R3	74,436,288	0.01	99.41	97.5	47.02	69,119,002(92.86%)	65,291,130(87.71%)

**Fig. 2:**
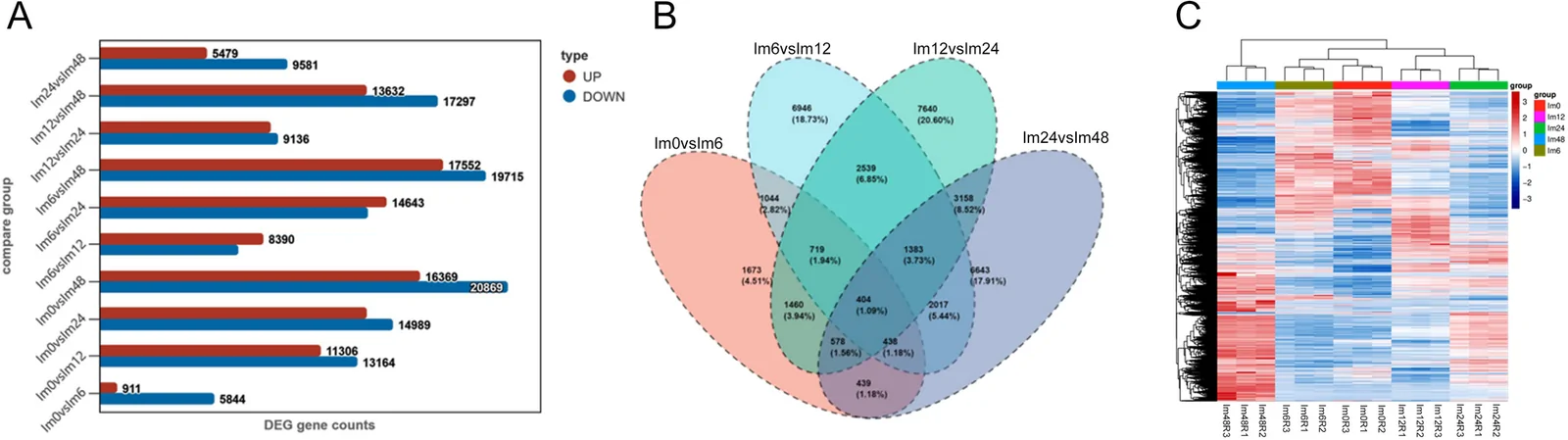
Analysis of gene expression in *B. napus* seeds at different imbibition stages. **A** Statistics of the number of significantly altered transcripts between successive stages in rapeseed seeds after imbibition. **B** Venn diagram analysis showing the overlap of differentially expressed genes (DEGs) between successive stages in rapeseed seeds after imbibition. **C** Hierarchical clustering heatmap of expressed genes at different stages in *B. napus* seeds after imbibition. the ordinate shows the FPKM-normalized values of differentially expressed genes; Redder colors indicate higher expression levels, while bluer colors indicate lower expression levels

In this study, differentially expressed genes (DEGs) were identified under the thresholds of |log₂(fold change)| ≥ 1 and adjusted *P*-value < 0.05. Two independent pairwise comparison strategies were applied to systematically resolve the dynamic transcriptional landscape during seed imbibition and germination.First, each imbibition time point was compared against the 0 h dry seed control. The total count of DEGs showed a continuous rising trend at 6 h, 12 h, 24 h and 48 h relative to Im0 (Fig. 2A, Table S1).Second, sequential pairwise comparisons between consecutive adjacent time points (Im6 h vs. Im0 h, Im12 h vs. Im6 h, Im24 h vs. Im12 h, Im48 h vs. Im24 h) were performed. DEGs derived from these four adjacent-stage comparisons were subjected to Venn analysis to quantify shared and unique transcripts (Fig. 2B). Only 404 DEGs were commonly detected across all four comparison groups, indicating that the vast majority of DEGs display obvious stage-specific expression features throughout germination. Hierarchical clustering of normalized expression values of all DEGs was conducted to display transcriptome patterns at different germination stages (Fig. 2C). Biological replicates of each time point clustered together. Im0 and Im6 samples were grouped into neighboring subclusters, while Im12, Im24 and Im48 samples formed separate distant branches.

**Fig. 3:**
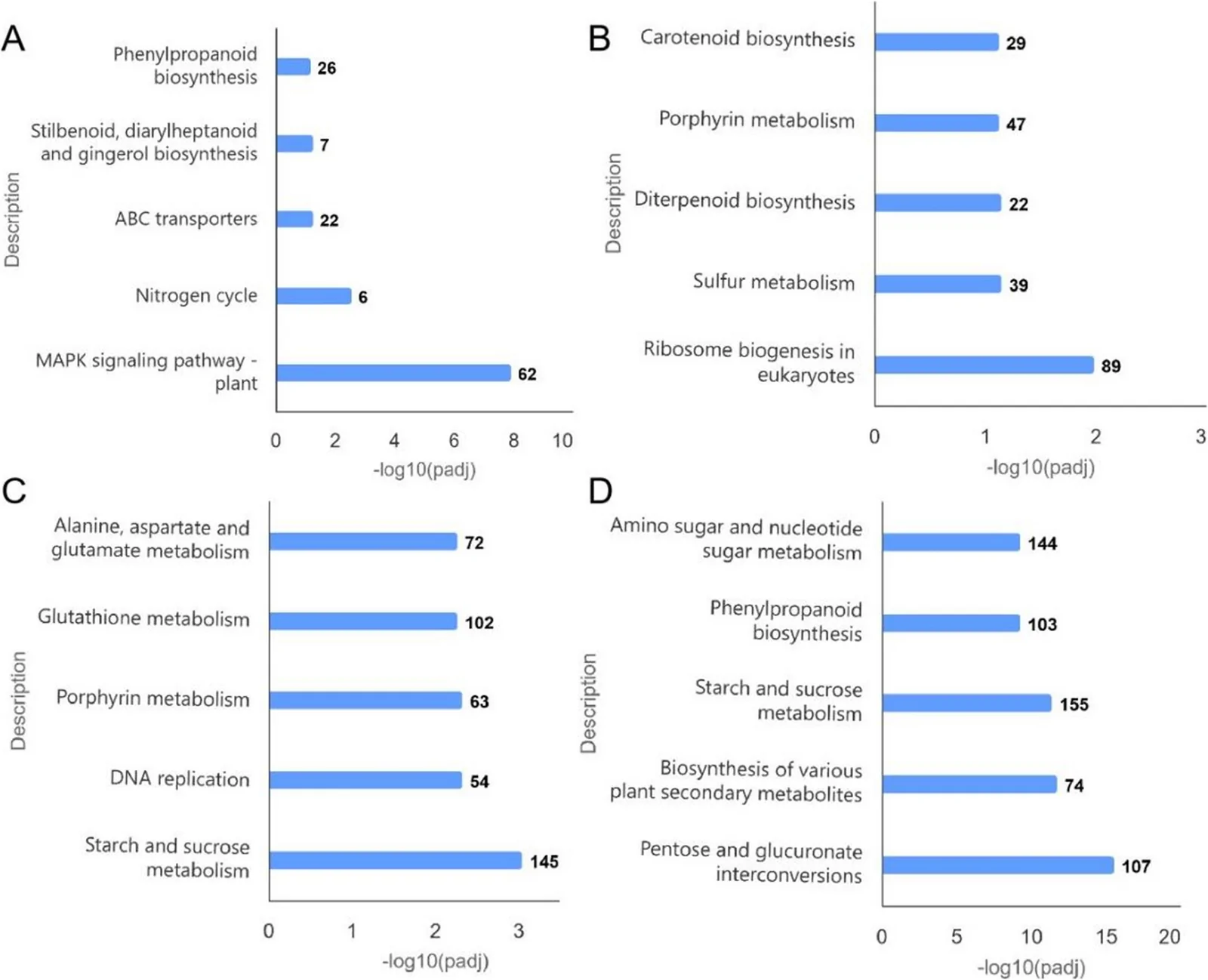
KEGG enrichment analysis of DEGs at different imbibition stages in *B. napus* seeds. **A**, **B**, **C**, and **D** represent the KEGG pathway enrichment results of DEGs at 6 h, 12 h, 24 h, and 48 h of seed imbibition, respectively

To reveal the biological roles of DEGs during seed imbibition and germination, we performed GO functional classification and KEGG pathway enrichment analysis for DEGs identified at each sampling stage. GO enrichment exhibited clear stage-dependent functional shifts consistent with germination progression (Figure S2). At 6 h, DEGs were predominantly enriched in biological processes (BP) related to water response, while cellular component (CC) terms centered on lipid droplet and cell wall (CW) structures that govern seed water absorption. At 12 h, top enriched BP terms focused on protein folding and DNA replication, reflecting robust preparation for de novo protein synthesis and cell cycle activation. At 24 h, lipid catabolism and energy metabolism constituted the core enriched biological processes, corresponding to the mobilization of seed storage lipids. At 48 h, DEGs were strongly enriched in hormone response pathways that drive cell elongation and post-germination growth. KEGG enrichment further corroborated this sequential transcriptional remodeling, At 6 h, plant MAPK signaling pathway showed the highest enrichment significance, acting as the core cascade transducing water uptake signals (Fig. 3A). At 12 h, ribosome biogenesis in eukaryotes ranked first, representing the dominant event of large-scale protein biosynthesis (Fig. 3B). At 24 h, starch and sucrose metabolism was the most prominent pathway, providing carbon and energy substrates for germinating seeds (Fig. 3C). At 48 h, starch and sucrose metabolism remained the top enriched pathway to sustain continuous energy supply for cell expansion and seedling establishment (Fig. 3D). Collectively, these enrichment results demonstrate a sequential transcriptional reprogramming cascade: early water signal perception, mid-stage protein biosynthesis and storage lipid mobilization, and late energy metabolism supporting seedling morphogenesis.

### Global dynamic changes of lipidome during seed imbibition and germination

**Fig. 4:**
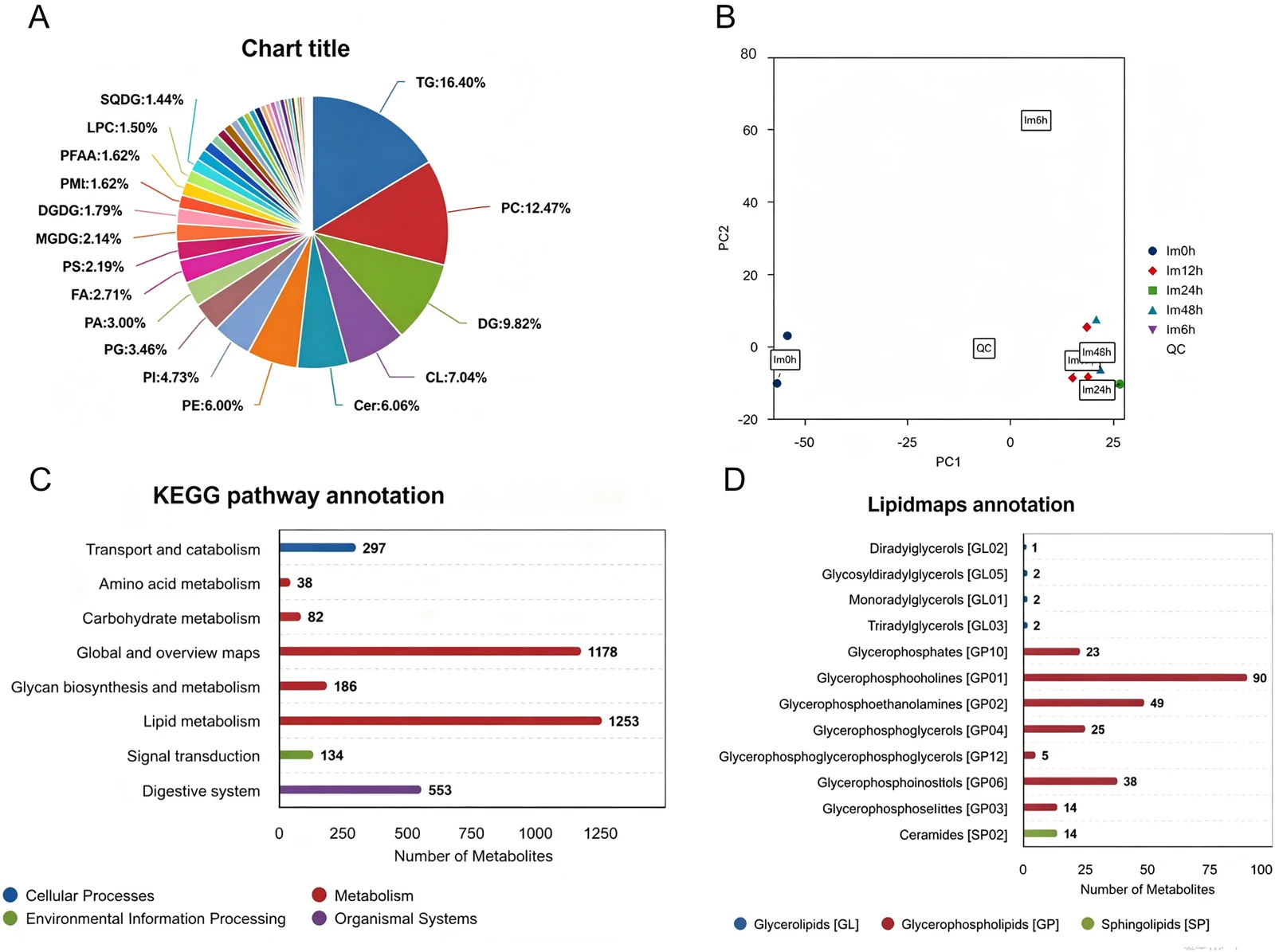
lipidomics analysis of *B. napus* seeds after imbibition. **A **Variations in lipid subclass levels in *B. napus* seeds during imbibition. **B **PCA analysis of all lipidomic samples from *B. napus* seeds after imbibition. **C **KEGG analysis of the identified lipid compounds. **D **Annotation of the eight major lipid classes and their subclasses for the identified lipids using the lipid maps database

To systematically characterize the lipidomic dynamics during *B. napus* seed imbibition and germination, lipidomic profiling was performed on the same seed samples as transcriptomic analysis, with three biological replicates per time point. A total of 1733 lipid molecules were identified across all samples, belonging to 15 major lipid classes, among which Triacylglycerol (TG) accounted for the highest proportion (16.40%), followed by Phosphatidylcholine (PC, 12.47%) and Diacylglycerol (DG, 9.82%) (Fig. 4A). PCA was conducted to evaluate the similarity of lipidomic profiles among samples, and the results showed that biological replicates of the same time point were closely clustered. Samples of different germination stages were clearly separated along PC1, which explained 46.62% of the total variation, suggesting significant temporal changes in lipid composition during seed imbibition and germination (Fig. 4B). Additionally, KEGG pathway annotation of all identified metabolites revealed that lipid metabolism was the most enriched pathway, containing 1253 annotated metabolites, followed by global and overview maps and the digestive system, highlighting the central role of lipid metabolism during seed imbibition and germination (Fig. 4C). Lipidmaps annotation demonstrated that glycerophosphocholines (GP01, PC) were the most abundant lipid subclass, followed by glycerophosphoethanolamines (GP02) and glycerophosphates (GP10) (Fig. 4D), Glycerolipidsand sphingolipids were also detected, confirming the dominance of phospholipids in membrane structure and the role of glycerolipids as storage and energy sources.

**Fig. 5:**
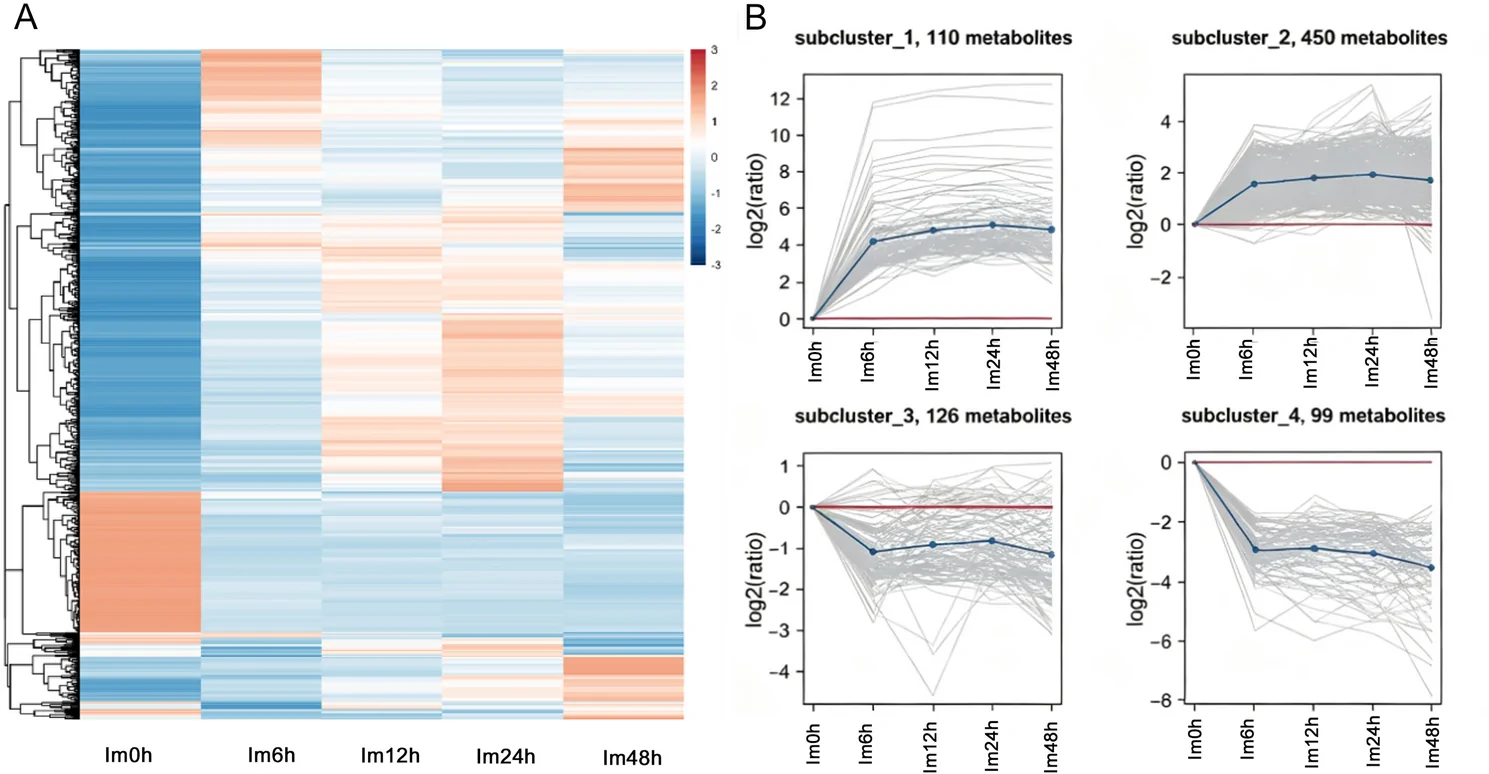
Differential analysis of lipid compounds in *B. napus* seeds at different imbibition stages. **A** Clustering analysis of all differential lipid compounds in *B. napus* seeds after imbibition. **B** K-means analysis of differential lipid compounds in *B. napus* seeds after imbibition

The hierarchical clustering heatmap (Fig. 5A) revealed distinct temporal patterns in lipid metabolite abundance across imbibition stages, with clear clusters of lipids showing increasing or decreasing abundance over time, reflecting dynamic lipid remodeling during seed imbibition and germination, Differential lipids are enriched in different imbibition stages (Table S2). K-means clustering (Fig. 5B, Table S3) further grouped these differential lipids into four subclusters: Subcluster 1 (110 metabolites, sustained upward trend throughout germination) was dominated by membrane lipids, with phospholipids as the largest class, followed by galactolipids and sphingolipid signaling lipids. Subcluster 2 (450 metabolites, peaking at 24 h before a slight decline) was the largest cluster, with phospholipids as the most abundant annotated class, alongside a high proportion of sphingolipid signaling lipids. Subcluster 3 (126 metabolites, sustained downward trend) was overwhelmingly dominated by phospholipids, with no other lipid class accounting for more than 10% of the cluster. Subcluster 4 (99 metabolites, continuous downward trend) was the most homogenous cluster, with phospholipids accounting for 89.9% (89 species) of all metabolites. These findings illustrate the coordinated, stage-specific lipid metabolic changes that underpin seed imbibition and germination, aligning closely with our transcriptomic observations.

**Fig. 6:**
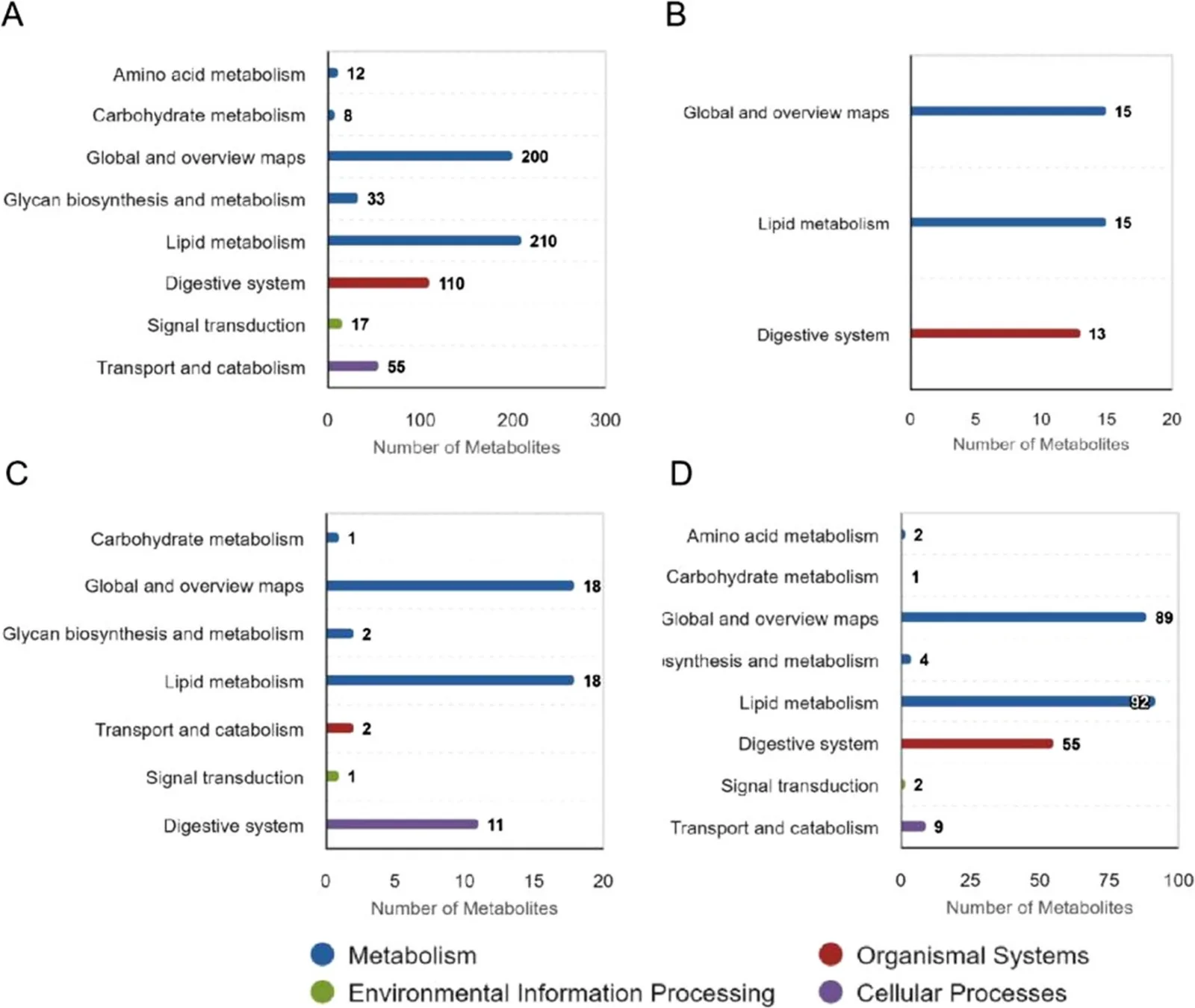
KEGG enrichment of differentially expressed lipids at different stages of seed imbibition. **A**, **B**, **C**, and **D** represent the KEGG pathway enrichment results of differentially expressed lipids at 6 h, 12 h, 24 h, and 48 h of seed imbibition, respectively

To dissect the temporal dynamics of lipid metabolism during seed imbibition and germination, we performed KEGG pathway annotation of lipid metabolites across four key time points. The analysis revealed a progressive activation of lipid metabolism: at 6 h (Fig. 6A), lipid metabolism was moderately represented (210 metabolites) alongside active glycan biosynthesis and core metabolic networks, reflecting the initial phase of water uptake and stress response; by 12 h (Fig. 6B), the enrichment signal of lipid metabolism pathways transiently weakened, with only 15 related differential lipids detected; at 24 h (Fig. 6C), lipid metabolism reactivated (18 metabolites) to support membrane repair and early growth; and by 48 h (Fig. 6D), it emerged as the most dominant pathway (92 metabolites), which aligns with transcriptomic observations of enhanced lipid catabolism to provide energy and metabolic precursors for the transition to active germination. This temporal progression demonstrates a tightly coordinated metabolic response that underpins seed imbibition and germination, with strong consistency between lipidomic and transcriptomic data.

### WGCNA analysis of DEGs

**Fig. 7:**
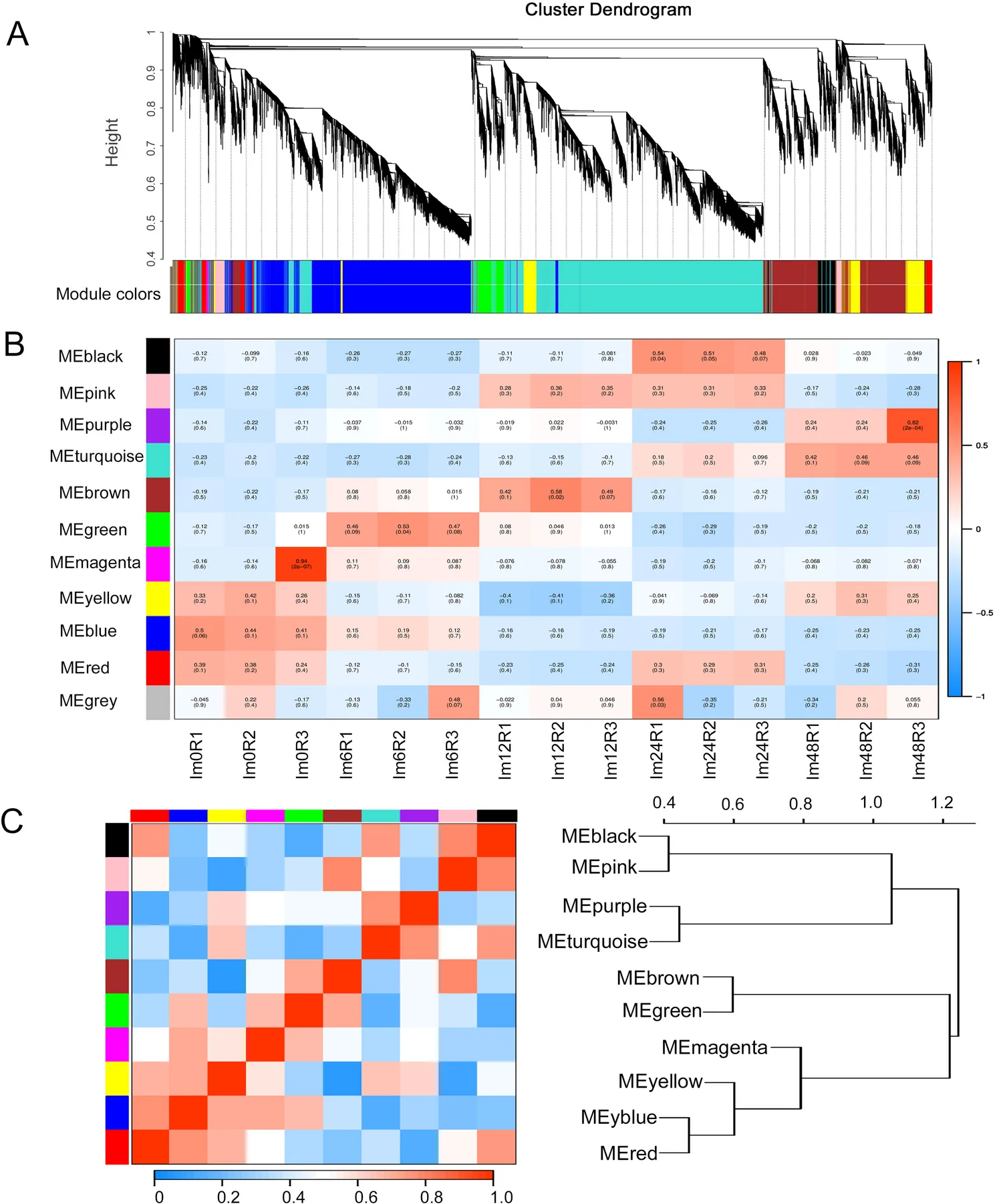
Analysis of WGCNA in *B. napus*. **A** Gene clustering dendrogram and module division. **B** Number of genes in each module and correlation coefficients between modules and lipid metabolic traits. Red and blue in the color scale represent positive and negative correlations, respectively. **C** Bar plot of module significance (MS) for lipid metabolism-related traits

To identify the potential regulatory network related to lipid metabolism and seed germination in *B. napus*, weighted gene co-expression network analysis (WGCNA) was performed based on the 34,689 high-expression genes retained after low-expression filtering. Genes with low expression were filtered out, and the remaining genes were used to construct a scale-free network. A total of 10 distinct co-expression modules were identified by hierarchical clustering (Fig. 7A). Module–trait correlation analysis showed that the MEblue module was strongly positively correlated with the catabolism of energy-storing substances such as lipids and carbohydrates, while The black module was associated with growth processes including membrane lipid biosynthesis (Fig. 7B). The inter-module correlation heatmap illustrated the relationships among the identified co-expression modules. Most modules exhibited weak to moderate correlations with one another, indicating that they participate in distinct biological processes (Fig. 7C).

### qRT-PCR validation of transcriptomic data

**Fig. 8:**
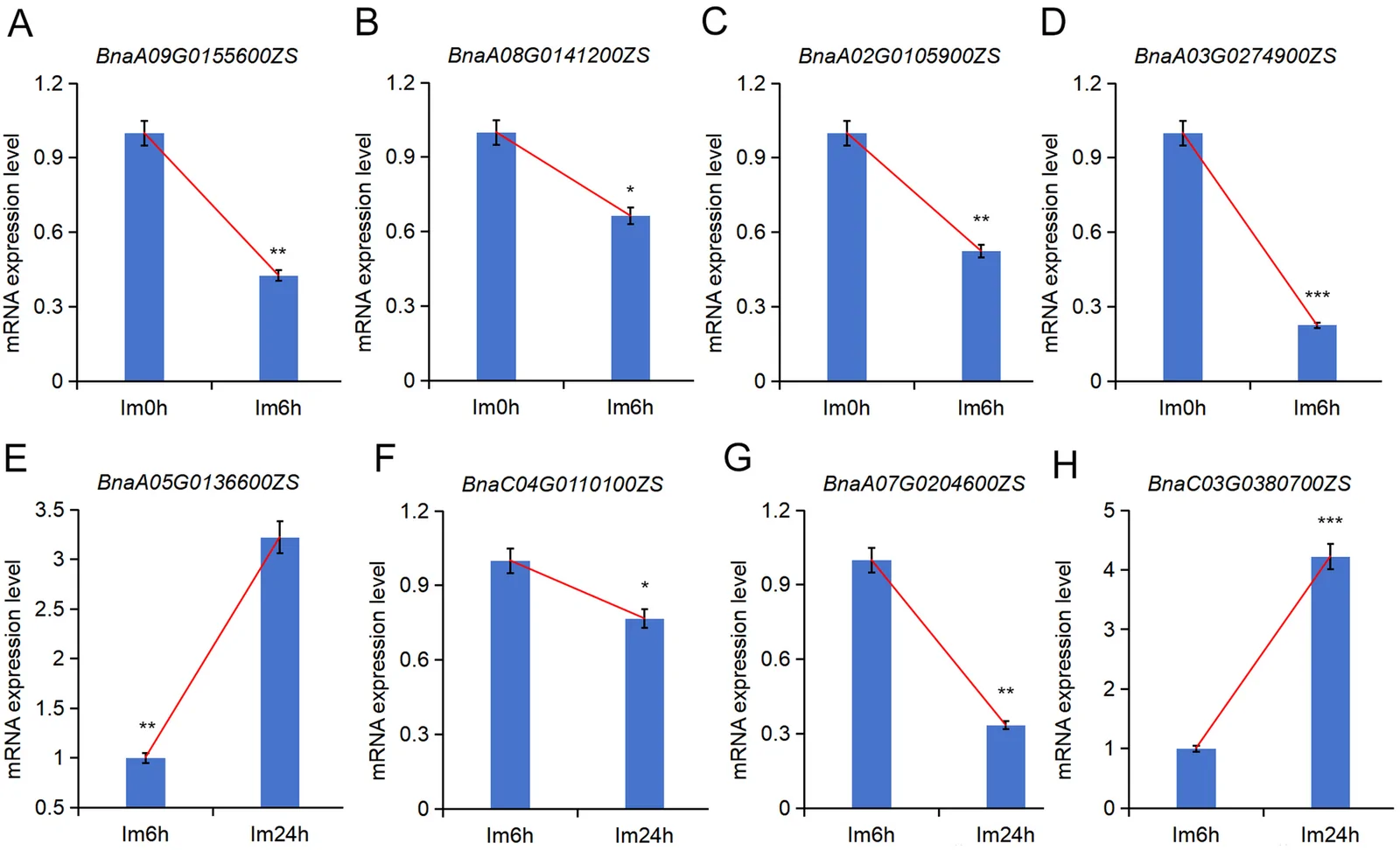
Expression level analysis of differentially expressed genes from the transcriptome in *B. napus* seeds after imbibition. Data are the means ± standard error (*n* = 3). Asterisks indicate the *P* value determined using a Student’s t test (*P < 0.05, **P < 0.01,***P < 0.001)

To confirm the credibility of transcriptome data, eight genes involved in lipid metabolism were selected for qRT-PCR detection (Fig. 8). These genes exhibited either significant upregulation or downregulation across imbibition stages, including those showing marked decreases in early imbibition (BnaA08G0155600ZS, BnaA03G0274900ZS) and those displaying increases over time (BnaA05G0136600ZS). The qRT-PCR expression profiles of these genes were fully consistent with their transcriptomic profiles, a strong correlation that supports the robustness of our transcriptomic analysis and the validity of the stage-specific transcriptional changes observed during seed imbibition.

## Discussion

### Early imbibition: a transcriptional preparation phase

The Early Stage of Seed Germination is an Active Transcriptional Preparation Stage [20]. In the rapid imbibition stage (0–6 h), DEGs identified by transcriptomic analysis were mainly enriched in biological processes such as water transport, response to water stimulus, and (CW) organization. The early imbibition stage already exhibits mild lipid metabolic remodeling, yet these lipid fluctuations are far less pronounced relative to the robust transcriptional activation of water transport, signal transduction and cellular structural adjustment. At this preparatory phase, transcriptional programs predominantly function to establish the physiological foundation required for subsequent lipid mobilization and radicle emergence, rather than driving vigorous lipid turnover. The activation of these DEGs may be closely associated with the rapid water absorption and swelling of *B. napus* seeds, a physiological process that is particularly critical for this oilseed crop [21, 22]. Unlike cereal crops, *B. napus* seed germination relies predominantly on stored lipids for energy supply, and the normal progression of early imbibition may directly determine whether subsequent lipid mobilization and metabolism can proceed smoothly [19, 23, 24]. Specifically, genes related to water transport, particularly aquaporins, may facilitate the rapid influx of water into seed cells during imbibition [25, 26]. This hydration process triggers a cascade of molecular events associated with the transition from quiescence to metabolic activation, including reactive oxygen species (ROS)-mediated signaling and hormonal rebalancing that promote dormancy release [27]. Concurrently, the increased cellular water content enables the rehydration-driven activation of hydrolytic enzymes, including lipases, which are prerequisites for the subsequent mobilization of stored lipids [28].

### Potential temporal regulation of TG degradation and energy supply conversion

Data from both transcriptomics and lipidomics indicated that there may be a precise temporal coordination between biochemical pathways such as β-oxidation and gluconeogenesis, manifested through stage-specific expression of peroxisomal ABC transporter 1 (PXA1), acyl-CoA oxidase 1/2 (ACX1/2) and phosphoenolpyruvate carboxykinase 1 (PCK1) during *B. napus* seed germination, and this mechanism might ensure the continuous supply of energy and carbon skeletons required for seed germination and seedling establishment. As the main storage lipid in *B. napus* seeds, triacylglycerols (TGs) serve as the core energy source during germination, the germination and early seedling establishment of *B. napus* are highly dependent on the efficient mobilization and metabolic conversion of TGs, making the putative precise temporal regulation of TG degradation particularly critical [29–31]. Our results showed that the stepwise degradation of TGs and their subsequent metabolic conversion are likely regulated by temporal programs at both the transcriptional and metabolic levels, which was well illustrated by the dynamic changes in KEGG pathway annotations across different imbibition stages.Specifically, at the early imbibition stage (0–6 h), transcriptomic analysis indicated that DEGs were mainly enriched in water transport and (CW) organization processes, while lipidomic profiles and KEGG annotations (Fig. 3A) showed only initial lipid metabolic remodeling without active TG degradation. This suggests that the transcriptional regulation at this stage prioritizes the establishment of a stable physiological environment for subsequent TG mobilization, rather than immediate energy supply conversion [32]. We identified distinct temporal dynamic patterns for storage TGs and structural membrane lipids throughout seed germination(Table S4). Massive breakdown of stored TGs provided energy and carbon skeletons for germination initiation, whereas progressive accumulation of phospholipids and galactolipids drove membrane reconstruction to support embryo cell division and elongation. This clear functional divergence between storage and membrane lipids precisely reflected the sequential physiological processes of lipid mobilization and membrane remodeling in imbibed rapeseed seeds.

### Enhancing seed germination quality to improve yield

Seed germination is the critical stage of the plant life cycle, directly determining crop emergence rate, seedling uniformity, and subsequent growth potential, which in turn affects final crop yield and quality [33, 34]. As a major global source of oil and protein, *B. napus* requires efficient germination and seedling establishment to ensure high yields and quality. This is crucial for maintaining a stable global oil supply and food security, particularly in the face of growing population pressure and environmental challenges [6, 35, 36]. From the perspective of rapeseed breeding practice, enhancing seed germination quality through molecular breeding strategies is a direct and effective approach to improve yield. The core genes and characteristic lipid metabolic markers identified by integrated multi-omics analysis can be used as potential molecular targets for breeding rapeseed varieties with superior germination performance [36]. Enhancement of seed germination quality can effectively elevate the field emergence rate, optimize the population structure, and improve the efficiency of resource utilization, thereby increasing the per unit area yield of *B. napus* without the need for cultivated land expansion.

## Conclusions

In this study, we conduct the first systematic integration of transcriptomic and lipidomic data focusing on dynamic changes during the germination stage to unravel the global molecular and metabolic landscapes of *B. napus* seeds at distinct imbibition phases, and define stage-specific expression signatures of core genes and lipid metabolites. This work extends our mechanistic knowledge of rapeseed germination regulatory pathways and supplies valuable theoretical foundations and candidate genes for high-quality, high-yield rapeseed breeding programs.

## Supplementary Information

Supplementary Material 1.

## Data Availability

All transcriptomic and lipidomic data generated in this study have been deposited in the National Center for Biotechnology Information (NCBI) public databases to ensure free and open access for all researchers. The transcriptomic sequencing data are available in the Sequence Read Archive (SRA) database under the accession number [SRR37226466, SRR37226465, SRR37226464, SRR37226463, SRR37226462, SRR37226461, SRR37226460, SRR37226459, SRR37226458, SRR37226457, SRR37226456, SRR37226455, SRR37226454, SRR37226453, SRR37226452]. All data can be accessed freely upon publication to facilitate related research on *B. napus* seed germination and lipid metabolism, and to provide support for rapeseed genetic improvement and breeding.

## Abbreviations

TG: Triacylglycerol
PC: Phosphatidylcholine
DG: Diacylglycerol
CL: Cardiolipin
Cer: Ceramide
PE: Phosphatidylethanolamine
PI: Phosphatidylinositol
PG: Phosphatidylglycerol
PA: Phosphatidic acid
FA: Fatty acid
PS: Phosphatidylserine
MGDG: Monogalactosyldiacylglycerol
DGDG: Digalactosyldiacylglycerol
PMt: Phosphatidylmethanol
PFAA: Perfluoroalkyl acid
LPC: Lysophosphatidylcholine
SQDG: Sulfoquinovosyldiacylglycerol
GO: Gene Ontology
KEGG: Kyoto Encyclopedia of Genes and Genomes
WGCNA: Weighted Gene Co-Expression Network Analysis
