# Supplementary material for: Temporal transcriptomic and lipidomic analysis reveals multi-omics dynamic profiles of B. napus seed germination
Source: BMC Genomics. 2026 Jul 20;27:855. doi: 10.1186/s12864-026-13202-y (PMC13670584; doi:10.1186/s12864-026-13202-y)
Supplement: Supplementary file 1 [file 12864_2026_13202_MOESM1_ESM.zip › Supplementary Files/Supplemental Figures.docx]

**Supplementary Figures**

A

B

**Figure S1.** Quality Control and Quantitative Analysis of ribonucleic acid sequencing(RNA-Seq) Sequencing Data. (A)Statistics of RNA-seq reads obtained from the 15 samples and mapping to the reference genome of *B. napus.* (B)Venn diagram of differentially expressed genes at different imbibition time points

C

D

A

B

**Figure S2. GO enrichment of differentially expressed lipids at different stages of seed imbibition.(A), (B), (C), and (D) represent the KEGG pathway enrichment results of differentially expressed lipids at 6 h, 12 h, 24 h, and 48 h of seed imbibition, respectively.**

A

A
